# Anticancer activity of drug-loaded calcium phosphate nanocomposites against human osteosarcoma

**DOI:** 10.1186/s40824-017-0099-1

**Published:** 2017-06-24

**Authors:** Kyoung Dan Son, Young-Jin Kim

**Affiliations:** Department of Biomedical Engineering, Catholic University of Daegu, Gyeongsan, 38430 Republic of Korea

**Keywords:** Calcium phosphate, Nanocomposite, Anticancer activity, Controlled release, Drug delivery

## Abstract

**Background:**

Calcium phosphate (CaP) based nanoparticles are considered to be ideal drug carriers for delivery of anticancer drugs because of their excellent biocompatibility and pH responsiveness. However, CaP nanoparticles have the problems of limited drug load capacity, initial burst release, and short-term release. Thus, we prepared the CaP nanocomposites containing anticancer drug such as caffeic acid (CA-NP), chlorogenic acid (CG-NP), or cisplatin (CP-NP) in the presence of alginate as a polymer template to control the release rate of drugs.

**Results:**

The drug-loaded CaP nanocomposites exhibited spherical shape with a size of under 100 nm and the size of nanocomposites was hardly affected by the addition of drug. UV-visible spectroscopic analysis confirmed the insertion of drug into the CaP nanocomposites. These nanocomposites showed an initial burst release of drug, followed by a prolonged release, in which the release profile of drugs was depended on the solution pH. In addition, the drug-loaded CaP nanocomposites revealed anticancer activity on human osteosarcoma in a manner dependent on concentration of drugs and time.

**Conclusions:**

The drug-loaded CaP nanocomposites can contribute to the development of a new generation of controlled drug release carriers for chemotherapy of cancers.

## Background

Nanoparticle-based drug delivery systems have emerged as one of the most promising means for improved cancer therapy [1]. Properly designed nanoparticles are able to separate the drugs from the blood stream and avoid renal clearance. These nanoparticles have promoted uptake of anticancer drugs into target sites and reduction of nonspecific damage to normal tissues caused by free drugs through an enhanced permeability and retention (EPR) effect [2]. In addition, nanoparticle systems have offered stable aqueous dispersion of drugs by surface modification and protected drugs from environmental degradation, resulting in enhanced anticancer activity [3].

Calcium phosphate (CaP) based nanoparticles are considered to be ideal drug carriers for delivery of anticancer drugs because of their excellent biocompatibility, bioactivity, and pH responsiveness [4]. Different from liposomes and polymer micelles, CaP nanoparticles are able to encapsulate various drugs in their rigid matrix to give an almost entire prevention of premature drug release in physiological condition of plasma (pH = 7.4) until they dissolve to calcium and phosphate ions in acidic environments such as in lysosomes (pH = 4.0–5.0) [1]. However, CaP nanoparticles have the problems of limited drug load capacity, initial burst release, and short-term release [5]. Therefore, the applications of CaP nanoparticles in the sustained drug delivery are limited, especially for small molecular and water-soluble drugs.

Recently, the combination of inorganic materials with polymers for the preparation of nanocomposites has been extensively investigated as an alternative in drug delivery system because it is an effective tool to improve the properties of polymer or inorganic nanoparticles [3–5]. The strong interfacial interactions between polymer and CaP via electrostatic interaction and hydrogen bonding could improve the mechanical properties, drug loading efficiency, and controlled drug release behavior of nanoparticles. While the release of drugs from CaP has been proved to be very fast because of the weak interaction between the drugs and CaP particles, the combination of CaP with polymer seems to be a practicable way to extend the release of drugs [6].

Caffeic acid is a plant derived polyphenolic compound that belongs to class hydroxycinnamic acid consisting of phenolic and acrylic function groups [7]. It has gained enormous attention because of its biological and pharmaceutical properties such as antioxidant, anti-inflammatory, and anticancer effects. Chlorogenic acid is an ester formed from caffeic acid and quinic acid, which contains both aliphatic and aromatic groups [8]. It shows the direct cytotoxic effects towards various human cancer cells and its biological activities have been also proved in vivo. However, the activities of these polyphenolic compounds are known to be limited for only few hours in a body.

Cisplatin is one of the most widely used platinum-based anticancer agents for treatment of a variety of human malignancies with the mechanism of inducing cellular apoptosis through disrupting DNA structure in cell nuclei [1]. Cisplatin forms crosslinks between purine bases within DNA and interferes with DNA repair mechanisms, causing DNA damages, subsequently inducing apoptosis in cancer cells [9]. However, the application of cisplatin in the clinic suffers from reduced efficacy and severe adverse side effects originating from its non-selective reactions with various biomolecules and nonspecific interactions with both normal and tumor tissues. Therefore, strategies for safer and more effective cisplatin therapy are desired.

With their high areas to volume ratio, polymer/inorganic material nanocomposites are expected to be excellent materials for biomedical applications [3–5]. In this study, we developed a simple wet chemical precipitation method for preparing CaP nanocomposites containing anticancer drug such as caffeic acid (CA-NP), chlorogenic acid (CG-NP), or cisplatin (CP-NP) in the presence of alginate as a polymer template. The prepared nanocomposites were systematically examined by considering their morphologies, chemical structures, crystalline phases, and drug loading capacities. The cumulative drug release profiles from the nanocomposites were investigated using in vitro release assay. Furthermore, the cytotoxicity of the drug-loaded CaP nanocomposites onto human osteosarcoma was evaluated via fluorescence microscopy and MTT assay.

## Methods

### Materials

Sodium alginate, calcium nitrate tetrahydrate (Ca(NO_3_)_2_·4H_2_O), ammonium phosphate dibasic ((NH_4_)_2_HPO_4_), caffeic acid, chlorogenic acid, cisplatin, ammonium hydroxide solution (NH_4_OH), and 3-(4,5-dimethylthiazol-2-yl)-2,5-diphenyltetrazoliumbromide (MTT) were purchased from Sigma-Aldrich Co. and were used without further purification. The human osteosarcoma cell line (MG-63) was obtained from the American Type Culture Collection (ATCC, USA). Dulbecco’s modified Eagle’s medium (DMEM), fetal bovine serum (FBS), penicillin–streptomycin, and Dulbecco’s phosphate-buffered saline (DPBS, pH 7.4) were obtained from Gibco BRL (USA). Slowfade gold antifade reagent and Live/Dead Viability/Cytotoxicity assay kit were purchased from Molecular probes (USA). Other reagents and solvents were commercially available and were used as received.

### Synthesis of nanocomposites

Drug-loaded CaP (CA-NP, CG-NP, and CP-NP) nanocomposites were synthesized as follows. 2 *w*/*v*% drug (caffeic acid, chlorogenic acid, or cisplatin) solution was first added to 300 mL of 0.05 *w*/*v*% sodium alginate solution for the formation of polymer–drug complex by hydrogen bonding and electrostatic interaction. Then, 35 mL of 0.1 M Ca(NO_3_)_2_·4H_2_O solution was added dropwise, and the pH was adjusted to 10 by the addition of 25 *w*/*v*% NH_4_OH. To this solution, a determined amount (Ca/*P* = 1.67) of 0.1 M (NH_4_)_2_HPO_4_ aqueous solution was added dropwise over a period of 2 h. The final concentration of drug in the reaction solutions was 3 wt% based on the weight of sodium alginate and CaP precursors (Ca(NO_3_)_2_·4H_2_O and (NH_4_)_2_HPO_4_). The mixture was stirred at 45 °C under air to induce the nucleation and growth of CaP crystals in the polymer–drug complexes. After 24 h, the resultant CaP nanocomposites isolated by tubular membrane dialysis in deionized water for 24 h, followed by lyophilization in vacuo. In addition, the drug-free CaP (SA-NP) nanocomposite was also synthesized under the same conditions to use as a reference standard.

### Characterization of nanocomposites

The morphologies of the drug-loaded CaP nanocomposites were observed by field-emission scanning electronic microscope (FE-SEM, JSM-6335F, JEOL, Japan) and transmission electron microscopy (TEM, H-7600, Hitachi, Japan). The average diameter of nanocomposites was determined by analyzing the SEM and TEM images with image analyzing software (Image-Pro Plus, Media Cybernetics Inc., USA). UV-visible spectra were recorded on a Hitachi U-2900 spectrophotometer (Japan). The attenuated total reflectance Fourier transform infrared (ATR–FTIR) spectra of the samples were obtained using an ALPHA spectrometer (Bruker Optics, USA) in the wavenumber range from 400 to 4000 cm^−1^. The crystalline phases of the nanocomposites were characterized by X-ray diffraction (XRD) carried out on a PANalytical X’Pert Pro X-ray diffractometer (The Netherlands) equipped with a Cu Kα radiation source operated at 40 kV and 30 mA. The samples were scanned over the 2*θ* range from 20 to 60° at a rate of 2°/min.

### In vitro release of drug from nanocomposites

Drug release studies were carried out in a thermostatical shaking incubator (BioShaker MRB-022UP, Taitec Co., Japan). A weighted amount (40 mg) of drug-loaded nanocomposites was first immersed into 40 mL of 0.01 M DPBS (pH = 4.5 or 7.4) at 37 °C. The supernatants were taken from the solution after 0.5, 1.5, 3, 5, 9, 18, and 36 h. The amount of released drug was determined by measuring the absorption of the samples at 285 nm for caffeic acid and 300 nm for chlorogenic acid and cisplatin using a UV-visible spectrophotometer. The percentage of released drug was then calculated based on the initial weight of drug incorporated in the nanocomposites.

### Cytotoxicity assay

To determine in vitro cytotoxicity of drug-loaded CaP nanocomposites, MG-63 cells (2 × 10^4^ cells/well) were seeded onto 48-well tissue culture plate and incubated for 24 h at 37 °C. Then, these cells were treated with nanocomposites (5–20 μg/mL of drug). The same amount of drug-free CaP (SA-NP) nanocomposite with CA-NP was used as a reference standard. After 48 h incubation, the cell viability was evaluated by the MTT assay. In addition, MG-63 cells were incubated with the nanocomposites containing 20 μg/mL of drug for 12 h–48 h and the cell viability was measured by the MTT assay. Qualitative cell viability assay was performed by using the LIVE/DEAD Viability/Cytotoxicity assay kit. The kit contains calcein AM and ethidium homodimer-1 (EthD-1), which identifies live versus dead cells on the basis of membrane integrity and esterase activity. Calcein AM stains live cells green, whereas EthD-1 stains dead cells red [10]. MG-63 cells (2 × 10^4^ cells/well) were seeded onto 8 well chamber slide and incubated for 24 h at 37 °C. Then, these cells were treated with the drug-loaded CaP nanocomposites (20 μg/mL of drug). After 24 and 48 h incubation, the cellular layers on the sample surfaces were treated for 10 min at 37 °C with 1 μM of calcein AM and 2 μM of EthD-1 to determine cell viability. Cells were finally observed using an inverted fluorescence microscope (Eclipse TS100, FITC-G2A filters, Nikon, Japan) equipped with a cooled CCD camera (DS-U2, Nikon, Japan) and with NIS-Elements Imaging Software.

### Statistical analysis

All data are expressed as means ± standard deviation. Statistical analyses were performed based on Student’s *t*-test. Comparison of different groups and significant difference were determined using SigmaPlot 10.0 (Systat Software, CA) where p* <0.05, p** <0.01, and p*** <0.001.

## Results and discussion

### Morphology of nanocomposites

CaP nanocomposites are well known as carriers for the transport of genes and drugs into cells because of enhanced bioavailability, drug loading capacity, and safety of drugs [3–5]. CaP is superior to other inorganic species such as silica in terms of biocompatibility because CaP is naturally found as the main mineral component in bone [11]. The polymer or CaP nanoparticles exhibited initially very fast drug release profile but polymer/CaP nanocomposites showed sustained release of drugs [6]. In the present study, the drug-free and drug-loaded CaP nanocomposites were prepared by precipitation method from water in the presence of polymer which stabilized the nanocomposites. The resulting nanocomposites were named as SA-NP, CA-NP, CG-NP, and CP-NP with different drug (Table 1).

**Table 1:** Characteristics of drug-loaded CaP nanocomposites

Sample	Drug feed ratio (wt%)^a^	Loading content (wt%)^b^	Mean diameter (nm)^c^
SA-NP	–	–	55.7 ± 6.9
CA-NP	3	1.0 ± 0.11	60.9 ± 7.7
CG-NP	3	1.3 ± 0.12	51.3 ± 5.1
CP-NP	3	1.7 ± 0.09	56.8 ± 6.5

^a^Weight feed ratio of drug to polymers and CaP precursors

^b^Weight content of drug in CaP nanocomposites, determined by UV–visible spectrophotometer

^c^Determined by analyzing the SEM and TEM images with image analyzing software

**Fig. 1:**
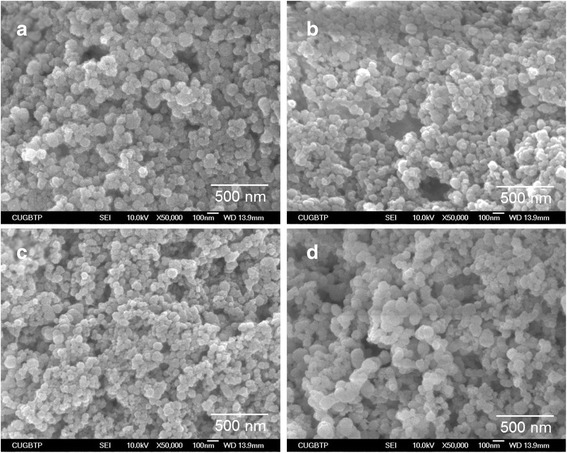
SEM micrographs of the drug-free and drug-loaded CaP nanocomposites: (**a**) SA-NP (drug free), (**b**) CA-NP (caffeic acid), (**c**) CG-NP (chlorogenic acid), and (**d**) CP-NP (cisplatin)

**Fig. 2:**
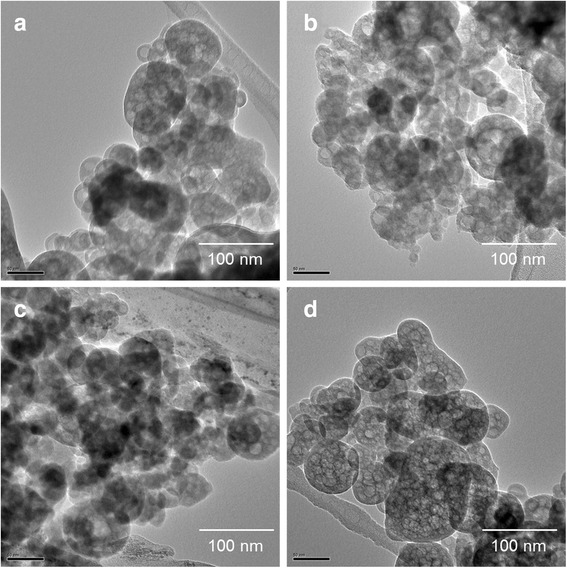
SEM micrographs of the drug-free and drug-loaded CaP nanocomposites: (**a**) SA-NP, (**b**) CA-NP, (**c**) CG-NP, and (**d**) CP-NP

Fig. 1 and Fig. 2 show the morphological structure of nanocomposites. All of the resulting nanocomposites exhibited spherical shape with a size of under 100 nm and the size of nanocomposites was hardly affected by the addition of drug. The average diameter of nanocomposites was 55.7 ± 6.9 nm for SA-NP, 60.9 ± 7.7 nm for CA-NP, 51.3 ± 5.1 nm for CG-NP, and 56.8 ± 6.5 nm for CP-NP as shown in Table 1. The existence of alginate can provide a lot of binding sites giving rise to the accumulation of drug molecules and Ca^2+^ ions due to the hydrogen bonding and ionic interaction. Therefore, their shape and size are consequence of the formation of a specific stereo-chemical arrangement and the charge distribution of reactive groups in alginate–drug and alginate–Ca^2+^ complexes [12]. These complexes can strongly interact with the surface of PO_4_^3−^ ions to nucleate the drug-loaded CaP nanocomposites. This means that the initial nucleation is preferentially caused at the positions of carboxyl groups, and the particle size is related to the nucleation and growth. Moreover, the drug-loaded CaP nanocomposites exhibited very narrow size distribution, suggesting that these nanocomposites are the optimum carriers for delivering drugs because they can easily pass through cell barriers and preferentially accumulate at the tumor sites based on the EPR effects.

### Physicochemical properties of nanoparticles

**Fig. 3:**
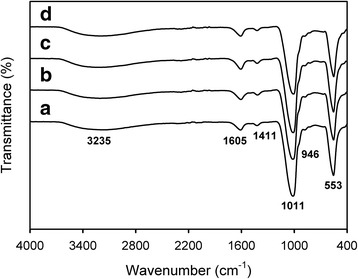
FT-IR spectra of (**a**) SA-NP, (**b**) CA-NP, (**c**) CG-NP, and (**d**) CP-NP

FT-IR analysis was carried out for identifying the functional groups present in the drug-loaded CaP nanocomposites, which, in turn, provided information about the constitution and phase composition of the products. All of the samples exhibited characteristic absorption bands for the vibrational modes of PO_4_^3−^ appeared at around 1011, 946, and 553 cm^−1^, and the bands at 3235 cm^−1^ associated with OH of alginate and absorbed H_2_O as shown in Fig. 3 [12]. In addition, the absorption bands for the asymmetric stretching mode of COO^−^ ion were observed at 1605 cm^−1^, which was probably ascribed to alginate. Furthermore, the band observed at 1411 cm^−1^ is attributed to the substitution of CO_2_^3−^ ions in the place of PO_4_^3−^ ions. These CO_2_^3−^ ions were formed by the reaction of CO_2_ present in the atmosphere with OH^−^ ions of reaction medium. However, the characteristic absorption bands ascribed to drug were not observed because of significant overlapping with the absorption bands of alginate and CaP, and thus the incorporation of drug was not clearly identified.

To confirm the incorporation of drug into the nanocomposites, the amount of drug loaded on the nanocomposites was determined by UV-visible spectroscopy. Among the samples, CP-NP exhibited higher drug loading content compared with the other nanocomposites because of strong electrostatic interaction between alginate and cisplatin (Table 1). The most effective method for incorporating cisplatin to the nanocomposites is based on the exchange of the chloride ion (Cl^−^) ligands of cisplatin with the oxygen donors such as carboxylates [1]. The drug loading content of nanocomposites was 1.0 ± 0.11 wt% for CA-NP, 1.3 ± 0.12 wt% for CG-NP, and 1.7 ± 0.09 wt% for CP-NP based on the weight of nanocomposites, as shown in Table 1.

**Fig. 4:**
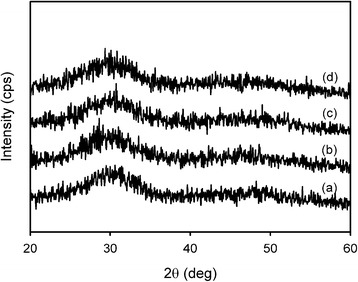
X-ray diffraction patterns of (**a**) SA-NP, (**b**) CA-NP, (**c**) CG-NP, and (**d**) CP-NP

Crystallographic analysis was performed using XRD to elucidate the change in crystalline phases of the drug-free (SA-NP) and drug-loaded CaP nanocomposites (Fig. 4). The XRD patterns of synthesized nanocomposites showed the peaks attributed to the amorphous CaP (ACP) phase [13]. All the peaks were broad diffraction peaks indicating the complex formation of CaP with amorphous polymer. In addition, the isomorphous substitution of PO_4_^3−^ by CO_2_^3−^ derived from the absorption of CO_2_ in the air during preparation process of the nanocomposites affected the formation of ACP phase. ACP can be easily biodegraded by cells and its degradation rate can be controlled. In addition, ACP can restrain aseptic inflammation, meaning that ACP can be an ideal candidate as a functional delivery system for chemotherapy of osteosarcoma [4].

### Drug release behaviors from nanocomposites

**Fig. 5:**
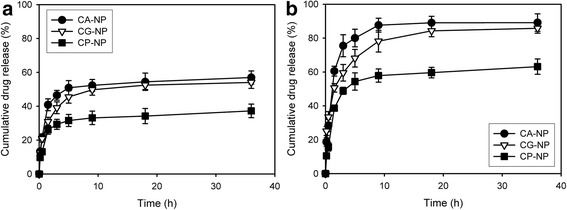
The cumulative release profiles of drugs from the nanocomposites in different pH of 0.01 M DPBS at 37 °C: (**a**) 7.4 and (**b**) 4.5

The physiological pH in the blood stream is 7.4 and the pH value of intracellular lysosome is 4.5 [1]. The ideal controlled drug delivery system requires the ability to suppress drug release during circulation in blood vessel but release the loaded drug in the targeted cells. In addition, for anticancer drugs, a desirable release profile should show a constant release rate with time. In our systems, an initial burst release of drug was observed, followed by a prolonged release as shown in Fig. 5. The initial burst release could be due to drug molecules entrapped into the shell wall by the hydrogen bonding and electrostatic interactions with carboxylates and hydroxyl groups [14].

Drugs were released faster from the nanocomposites at pH 4.5 than at pH 7.4 in the DPBS solution because of pH-dependent dissolution of CaP. This dissolution of CaP layers certainly removed the diffusion barrier, thereby enhancing the drug release rate. It is well known that the release behaviors of drug molecules on CaP nanoparticles are complicated and normally depend on the equilibrium concentration of the drug, pH, and other factors of the surrounded environment [5]. In addition, the release rate of cisplatin was slower comparing to the other drugs. As described above, cisplatin could be conjugated to carboxylates of alginate and achieved binding affinity to Ca^2+^ ions through the substitution of its Cl^−^ ligands with PO_4_^3−^ ions, inducing the controlled release of cisplatin.

### In vitro anticancer activity of nanocomposites

**Fig. 6:**
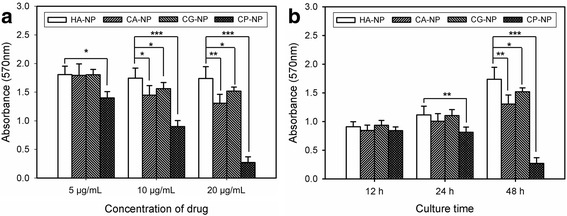
In vitro anticancer activity of the drug-loaded CaP nanocomposites on MG-63 cells. The cells were incubated (**a**) with different concentration of nanocomposites (5–20 μg/mL of drug) for 48 h and (**b**) with nanocomposites containing 20 μg/mL of drug for different culture time (*n* = 5). The same amount of SA-NP with CA-NP was used as a reference standard. (p* ˂0.05, p** ˂0.01, p*** ˂0.001)

To evaluate the potential of the nanocomposite drug delivery system in the application of cancer therapy, the drug-loaded CaP nanocomposites were used to investigate their cytotoxicity onto human osteosarcoma cell line (MG-63). The in vitro anticancer activity of the nanocomposites was estimated by MTT assay using the drug-free CaP (SA-NP) nanocomposite as a reference standard (Fig. 6). The data indicated no effect of SA-NO on the viability of MG-63. However, the drug-loaded CaP nanocomposites showed anticancer activity on MG-63 in a manner dependent on concentration of drugs. In particular, cisplatin-loaded CaP (CP-NP) nanocomposite exhibited higher anticancer activity than that of other nanocomposites. Moreover, cell viability was gradually suppressed by the use of drug-loaded CaP nanocomposites compared with SA-NP during the incubation period. These results suggest that the encapsulation of drugs in CaP nanocomposites can control the drug release rate and improve the anticancer activity of drugs on human cancer cells.

**Fig. 7:**
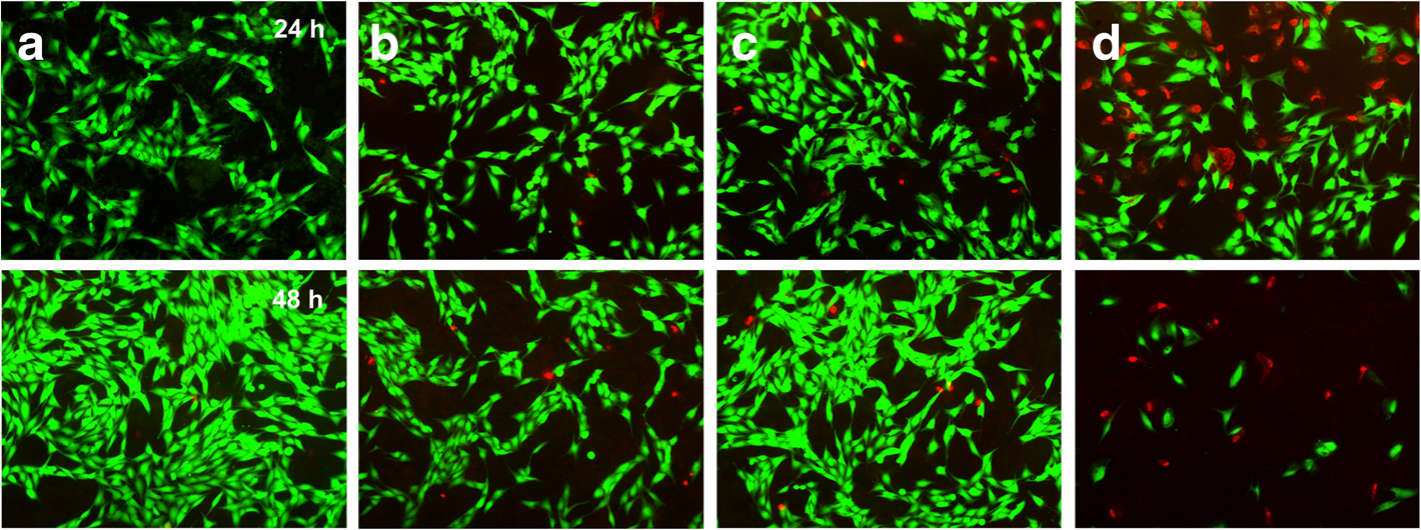
Live/Dead fluorescence microscopy images of MG-63 cells stained with calcein-AM (*green*) and EthD-1 (*red*) in the presence of (**a**) SA-NP, (**b**) CA-NP, (**c**) CG-NP, and (**d**) CP-NP. The same amount of SA-NP with CA-NP was used as a reference standard

Cancer cell viability after the treatment with the drug-loaded CaP nanocomposites was further confirmed by a fluorescence staining study with calcein AM (green fluorescence) and EthD-1 (red fluorescence) to distinguish the live and dead cells. As shown in Fig. 7, MG-63 cells treated with SA-NP showed only green fluorescence even though after incubation for 48 h, indicating live cells. However, red fluorescence appeared and green fluorescence decreased by the treatment of MG-63 cells with the drug-loaded CaP nanocomposites due to the cell death. This is in good agreement with the MTT assay result of the drug-loaded CaP nanocomposites.

## Conclusion

The engineered nanocomposites have received attention as a possible means of encapsulating and delivering anticancer drugs. In particular, the combination of polymer and inorganic material seems to be a practicable way to prolong the drug release. In the present study, a simple reaction for the preparation of drug-loaded CaP nanocomposites was successfully developed by rapid precipitation from water in the presence of polymer template which stabilized the nanocomposites. The resulting nanocomposites had spherical structure and very narrow size distribution. The drug-loaded CaP nanocomposites showed slow, long-term, and controlled release rate in DPBS. In addition, drugs were released faster from the nanocomposites at pH 4.5 than at pH 7.4 because of pH-dependent dissolution of CaP. The drug-loaded CaP nanocomposites revealed anticancer activity on MG-63 in a manner dependent on concentration of drugs and time. In particular, cisplatin-loaded CP-NP nanocomposite exhibited higher anticancer activity than that of other nanocomposites. Based on these results, the drug-loaded CaP nanocomposites can contribute to the development of a new generation of controlled drug release carriers for chemotherapy of cancers.

## Abbreviations

ACP: Amorphous calcium phosphate
ATR–FTIR: Attenuated total reflectance Fourier transform infrared
CaP: Calcium phosphate
CCD: Charge-coupled device
DMEM: Dulbecco’s modified Eagle’s medium
DNA: Deoxyribonucleic acid
DPBS: Dulbecco’s phosphate-buffered saline
EPR: Enhanced permeability and retention
EthD-1: Ethidium homodimer-1
FBS: Fetal bovine serum
FE-SEM: Field-emission scanning electronic microscope
MTT: 3-(4,5-dimethylthiazol-2-yl)-2,5-diphenyltetrazoliumbromide
TEM: Transmission electron microscopy
UV: Ultraviolet
XRD: X-ray diffraction
